# Gender inequalities in high psychological distress vary across European regions and occupational subgroups

**DOI:** 10.1038/s41598-026-54327-0

**Published:** 2026-05-28

**Authors:** Julia Grasshoff, Batoul Safieddine, Stefanie Sperlich, Johannes Beller

**Affiliations:** 1 Department for Medical Sociology, Hannover Medical School, Hanover, Germany; 2 Department for Medical Sociology, Medical University Lausitz - Carl Thiem, Cottbus, Germany

**Keywords:** Psychology, Environmental social sciences

## Abstract

Gender inequalities in psychological distress have been found in various populations, including occupational groups and countries. There is a lack of studies that compare regions in a uniform operationalisation of distress and integrate occupational groups. 67,641 participants from 36 countries of the European Working Conditions Survey 2021 were analysed. High psychological distress was operationalized using the WHO-5 Well-Being Index. Using predicted probabilities and multilevel regression analysis, gender inequalities between countries, regions and occupational subgroups were calculated. Across most countries, women reported higher psychological distress. The magnitude of gender differences varied between countries, regions and occupations. However, overlapping confidence intervals limit the conclusiveness of the results. When stratifying for both region and occupation type, in the Southern European region and in blue-collar low-skilled occupations gender inequalities were highest. In the significant interaction model, white-collar occupations in Western Europe and blue-collar low-skilled occupations in Southern Europe had the highest gender inequalities. This study demonstrates heterogeneity in the magnitude of gender differences in high psychological distress across Europe and occupational groups. White-collar high-skilled occupations in Western Europe and blue-collar low-skilled occupations in Southern Europe were identified as vulnerable groups. The findings underscore the importance of considering regional patterns when addressing gender-based mental health inequalities in the European workforce.

## Introduction

### Psychological distress and work

Psychological distress can be conceptualized as an umbrella term describing multiple mental and physical symptoms associated with fluctuations of mood such as sadness, fatigue, helplessness or irritability. It impacts quality of life and workplace productivity. At the same time, it can be increased by certain occupational demands themselves. It is prevalent among employees and has been identified as a predictor of absenteeism and disability^1,2^. Followed by productivity losses due to absence and reduced work performance, psychological distress imposes a socioeconomic burden on employers and the broader economy.

### Gender inequalities in psychological distress

Gender disparities in psychological distress represent a significant concern in health research, with women consistently exhibiting a greater burden than men. In a large-scale Finnish sample of full-time employees across various occupations, 11.0% of women, compared to 8.8% of men, reported experiencing symptoms of psychological distress such as nervousness, feelings of dejection, or difficulty being consoled^3^. Similarly, a large-scale German survey found that employed women reported a significantly higher number of complaints while working such as insomnia, fatigue, irritability or low mood. This was found in different occupational subgroups when stratifying into blue collar and white collar and high skilled and low skilled occupations^4^.

Additionally, employment in female-dominated occupations has been associated with a higher prevalence of psychosomatic symptoms, and women are subject to distinct psychosocial work demands. For instance, a German study by Hünefeld and Dötsch^5^ found that employees in female-dominated professions reported more psychosomatic complaints and greater emotional exhaustion compared to those in male-dominated or gender-balanced fields. The authors suggested that the specific demands inherent in female-dominated roles may contribute to increased psychological distress. Similarly, a meta-analysis by Campos-Serna et al.^6^ found that women generally face greater job insecurity, lower job control, and poorer working conditions, whereas men are more likely to experience effort–reward imbalances and longer working hours. The analysis concluded that women’s work-related demands may be more predictive of psychological distress than those of men; however, most included studies did not account for occupational type, leaving it unclear whether these findings stem from the nature of female-dominated occupations or from being female in the workplace.

One prominent framework for understanding these disparities is the concept of the gender care gap. Despite increased female participation in the labour market, women in Europe continue to shoulder most unpaid care responsibilities such as childcare, looking after relatives and friends, household chores or grocery shopping. In a time trend analysis of European countries between the 2000s and 2010, Campaña et al.^7^ found a decreasing but enduring gap between men and women’s unpaid working hours. The gender care gap is connected to heightened psychological distress among employed women. This was summarized in a scoping review in Gencer et al.^8^, describing a slightly decreasing gender gap during the COVID19-pandemic compared to the years before and at the same time women being mentally more affected by an increase of care responsibilities during the pandemic.

In summary, gender disparities in psychological distress are consistently documented across various occupations. However, a common methodological limitation is the lack of stratification by occupational group when comparing genders. Many studies either focus exclusively on a single occupation or fail to differentiate between occupations when analysing gender-based outcomes.

### Gender inequalities in different countries

The gender disparities in psychological distress described above have been consistently observed across various national contexts, including studies conducted in Finland^3^, Sweden^9^, and Germany^4,5^, as well as in systematic reviews and cross-national analyses^10,11^, all of which report a greater burden among women.

While gender inequalities are evident across countries, geographical variation in their magnitude has also been documented using depression scales which can be seen as a tool to operationalize psychological distress focusing on a potentially pathological extent. In a large-scale European study of adults aged 60–85, Schmitz and Brandt^12^ found that gender differences in depressive symptoms were relatively smaller in Northern, some Eastern, and most Western European countries, whereas Southern European countries exhibited more pronounced gender gaps. Similarly, Van de Velde et al.^10^ identified a regional pattern, with the largest gender disparities in depression rates found in Eastern and Southern Europe.

Herlitz et al.^11^ summarized in a review about the “gender equality paradox”, that in countries with present gender inequalities, the gender inequalities in negative emotion were bigger in countries with higher living conditions (higher GDP, years of education).

However, it is important to note that these studies focused on general populations rather than employed individuals, limiting the ability to draw conclusions about the role of occupational factors in shaping gender differences in psychological distress.

### Occupational inequalities in psychological distress and their interaction with gender

Levels of psychological distress have been shown to vary across occupational groups. The literature on health inequalities includes comparisons between occupational groups, with a common categorization being that of blue-collar and white-collar occupations. White-collar occupations refer to non-manual jobs, often of an administrative or organizational nature. Blue-collar refers to manual occupations such as skilled trades. A more nuanced distinction is based on the level of qualification required to perform the job: high-skilled vs. low-skilled. In a Finnish study, Lahti et al.^13^ found that high-skilled and white-collar workers reported the highest levels of emotional exhaustion, a key symptom of psychological distress, while low-skilled and blue-collar workers had the lowest levels. The authors describe how occupational subgroups differ in workplace requirements and those interacting with symptoms of psychological distress. Higher skilled occupations were associated with higher job control and higher job demands – the first may protect against exhaustion, the second increase exhaustion. Lower skilled and blue-collar coded occupations in turn were associated with lower job control. A Swiss study by Hämmig and Bauer^14^ reported that employees in occupations with white-collar characteristics experienced higher levels of strong distress. They describe differing characteristics of blue-collar work (such as such as monotony and low autonomy at work or low flexibility of working hours) and white-collar work (such as high time pressure, regular overtime, frequent interruptions or poor work-life compatibility). Similarly, in a German sample, gender disparities in distress, operationalized through psychosomatic complaints, were observed across various occupational subgroups—including blue- and white-collar as well as high- and low-skilled roles—with the most pronounced gender differences occurring among high-skilled white-collar workers^4^.

Despite these findings, research specifically examining gender inequalities in psychological distress within distinct occupational subgroups remains limited.

In conclusion, occupational variation in psychological distress exists and interacts with gender. However, there is a lack of cross-national research that compares gender disparities within occupational subgroups using consistent, validated measures of psychological distress. To identify and understand potential patterns more effectively, future analyses should stratify data by occupational group and gender, applying uniform measures across countries and regions. The present article aims to address this gap by integrating these dimensions into a comprehensive analysis.

### Research questions

We ask:

1. To what extent do gender inequalities in high psychological distress differ across individual European countries, and how pronounced are these differences?
2. How do gender inequalities in high psychological distress vary across broader European regions (Northern, Southern, Eastern, and Western Europe)?
3. How do gender inequalities in high psychological distress manifest across different occupational subgroups (i.e., high-skilled white-collar, low-skilled white-collar, high-skilled blue-collar, low-skilled blue-collar)?
4. Are there consistent regional patterns in the manifestation of gender inequalities in psychological distress across occupational subgroups within Europe?

The study has an exploratory nature due to little research with our focus. Nevertheless, based on existing findings, we anticipate the presence of gender inequalities in psychological distress across all European countries, with the most pronounced disparities expected among high-skilled white-collar workers. The study and research questions were not pre-registered.

## Methods

The dataset employed in this study originates from the 2021 wave of the European Working Conditions Survey (EWCS), commissioned by Eurofound and conducted by Ipsos. The EWCS aims to collect comparative data on the quality of working life across European Union member states, as well as candidate and potential candidate countries. Sample sizes varied between countries due to differences in budget allocation. Eurofound encourages researchers and policymakers to use the dataset to identify at-risk groups, monitor emerging issues, and track long-term trends in working conditions.

Participants were eligible if they were at least 16 years old and had engaged in paid employment for a minimum of one hour during the week of the survey. Data collection was conducted using a computer-assisted telephone interviewing (CATI) method, employing random direct dialling of mobile (cell) phone numbers. The overall response rate was 5%. According to Eurofound, there were notable sampling imbalances: in several Northwestern European countries, younger individuals were underrepresented while older respondents were overrepresented; the opposite pattern was observed in many Southern and Eastern European countries. Furthermore, high-skilled and white-collar occupations were generally overrepresented, whereas blue-collar occupations were underrepresented. These imbalances are attributed in part to differing working conditions during the COVID-19 pandemic. Greater phone accessibility among remote workers versus those unable to work due to lockdowns or job disruptions, or exclusion based on survey eligibility criteria may have influenced reachability. Additionally, workers with children were frequently underrepresented.

To address these limitations, the analyses applied sampling weights provided by Eurofound and described in their technical report^15^. These weights were primarily derived from Eurostat data, though in cases where Eurostat data were incomplete, information from national statistical institutes was requested and incorporated. The use of these weights enhanced the representativeness of the data with respect to key demographic and labour market variables, including sex, age, occupation, and region.

### Description of variables

To operationalize psychological distress, the five-item World Health Organization Well-Being Index (WHO-5) was employed. The WHO-5 assesses well-being and psychological distress over a two-week period, aligning with the World Health Organization’s holistic definition of health as a state of complete physical, mental, and social well-being rather than merely the absence of disease or infirmity. Participants responded to five items phrased as: “Over the last two weeks, how often have you been feeling …” followed by “… cheerful and in good spirits?”, “… calm and relaxed?”, “… active and vigorous?”, “… fresh and rested when you woke up?”, and “… that your daily life has been filled with things that interest you?”. Responses were recorded on a six-point Likert scale ranging from 0 (“At no time”) to 5 (“All of the time”), resulting in a raw WHO-5 sum score between 0 and 25. To operationalize high psychological distress (nominal: yes/no), a cut-off of 50% of the transformed WHO-5 score was applied, using the formula WHO5 raw score × 4 to yield a value between 0 and 100 as recommended by Topp et al.^16^. The threshold of 50% (or raw score of 12.5 points) has been validated as an appropriate screening criterion for depression risk^16,17^. As the data originates from standardized interviews of non-health-professionals, a valid diagnosis of clinical depression cannot be made. This kind of data would need a clinician to do the interview, expanding standardized questions with their trained, clinical impression. Alternatively, health insurance records could provide diagnostic information, but such data are not available in our dataset. Thus, we cannot determine whether participants meet diagnostic criteria. Operationalizing high psychological distress as ‘risk of depression’ using the given formula is therefore the most appropriate approach.

The variable “gender” was dichotomized into male and female categories. Approximately 0.3% of participants identified as “other”; due to the small sample size and the inappropriateness of assigning these cases to either male or female categories, they were excluded from the analysis.

Participants’ self-reported occupations were recoded into the variable “collar” based on the International Standard Classification of Occupations (ISCO88), a system demonstrated to have high reliability^18^. Using skill level (complexity and range of tasks) and skill specialization (field of work, tools, and types of goods or services), occupations were grouped into four superordinate categories: “white-collar high-skilled” (e.g., legislators, senior officials, managers, professionals, technicians, associate professionals); “white-collar low-skilled” (e.g., clerks, service workers, shop and market sales workers); “blue-collar high-skilled” (e.g., skilled agricultural and fishery workers, craft and related trades workers); and “blue-collar low-skilled” (e.g., plant and machine operators, assemblers, elementary occupations). Members of the armed forces were excluded. This classification has been previously applied by studies using data from Eurofound and the European Commission Joint Research Centre^19,20^. In the analyses, occupational group was treated as a nominal variable.

Covariates included age (numeric) and usual weekly working hours (numeric). As described in a European wide comparison^21^ or a Japanese study including multiple birth cohorts and data waves^22^, symptoms of depression and psychological distress vary by age with increasing burden with older age. Several studies have identified working more than 35 h per week as a predictor of poorer well-being and increased depressive symptoms^23,24^, though the strength and pattern of this association vary by region and gender in Europe-wide analyses using the European Working Conditions Survey^25^. With regard to the influence of the variables, these should be controlled in the analyses.

The analysis encompassed 36 European countries. To address variability in sample sizes when considering both country and occupational subgroup, countries were grouped into the four regions Western, Southern, Eastern, and Northern Europe, following the United Nations geoscheme for Europe^26^. The regional classification was as follows: Western Europe included Austria, Belgium, Germany, France, Luxembourg, Netherlands, and Switzerland; Eastern Europe included Bulgaria, Czechia, Estonia, Hungary, Poland, Romania, Slovakia, Kosovo, and Montenegro; Southern Europe included Cyprus, Greece, Spain, Croatia, Italy, Malta, Portugal, North Macedonia, Slovenia, Serbia, Albania, and Bosnia & Herzegovina; Northern Europe included Denmark, Finland, Ireland, Sweden, United Kingdom, Norway, Lithuania, and Latvia.

The initial dataset comprised 71,758 cases. After deletion of cases with missing data on relevant variables (5.74%), 67,641 participants remained for analysis.

### Data analysis

First, descriptive statistics (means, standard deviations, and percentages) were calculated to characterize the sample.

To address Research Question 1, predicted probabilities estimating adjusted prevalences of high psychological distress were computed for each country separately using logistic regression analyses. Due to large sample sizes per country, intercepts and slopes were independent. In these models, the binary outcome variable was “high psychological distress”, with gender as a categorical predictor. Age and usual weekly working hours were included as covariates. Predicted probabilities were derived post-estimation to adjust for differences in age and working hours distributions across genders. Odds ratios (ORs) with 95% confidence intervals (CIs) were calculated for each country based on the same logistic regression model.

For Research Questions 2 through 4, multilevel logistic regression analyses were conducted to examine gender inequalities in high psychological distress across countries, accounting for the hierarchical data structure where individuals (level 1) were nested within regions (level 2). This approach accounts for the non-independence of observations and allows for robust effect estimation. The primary outcome variable was the binary indicator of high psychological distress, and the main predictor was gender, with age and weekly working hours included as covariates. For multilevel regression analysis, we included random intercept and fixed slope.

An initial overall model including all occupational groups was estimated to assess the general gender effect across European regions. Subsequent analyses stratified the sample by occupational subgroup. For each model, odds ratios with 95% confidence intervals quantified the association between gender and psychological distress. Interaction terms between gender, region, and occupational group were first included to test whether gender inequalities varied by these factors. Lastly, a follow-up model without interaction terms was run.

All analyses incorporated the survey weighting variable provided with the dataset. Sensitivity analyses replicating key models without weighting and excluding covariates were performed, and corresponding results are presented in the Appendix. The multilevel logistic regression analyses were performed using R software. All other analyses were performed using Stata/MP 15.1 software.

## Results

### Descriptive variables

As shown in Table 1, females comprised 47.91% of the sample. The mean age was 42.31 years (SD = 12.05) for women and 41.51 years (SD = 12.46) for men. The prevalence of high psychological distress – defined as scoring below 50% of the maximum WHO-5 score – was 19.46% among men and 25.68% among women.

**Table 1:** Descriptive data.

	Men	Women
Number of participants (*N*/ %)	35,236/52.09%	32,405/47.91%
Age in years mean (SD)	41.51 (12.46)	42.31 (12.05)
Usual hours worked per week Mean (SD)	41.95 (12.74)	37.43 (12.30)
Proportion of subgroup having high psychological distress	19.46%	25.68%

### Absolute levels of high psychological distress in different European countries

As shown in Table 2, Kosovo had the lowest predicted probability of high psychological distress, with 7.59% for men and 10.22% for women. Furthermore, Romania, Denmark, and Finland had the lowest values in Europe for both gender groups.

The highest predicted probabilities were found in the United Kingdom, with 36.14% for men and 43.17% for women. Among men, this was followed by Slovakia, Greece, Cyprus, and Serbia (in descending order). Among women, the countries following the UK were Slovakia, Czechia, Cyprus, and Serbia. Results were robust to excluding the covariates in a sensitivity analysis with nearly identical results (see in the Appendix Table 4). However, wide confidence intervals and varying sample sizes limit the ability to make definitive statistical comparisons between individual countries, particularly regarding significance.

**Table 2:** Predicted probabilities of high psychological distress by country and gender.

Country	PP Men	95% CI Men	Country	PP Women	95% CI Women
Kosovo	07.59	[0.05, 0.10]	Kosovo	10.22	[0.06, 0.15]
Romania	09.98	[0.08, 0.12]	Romania	13.51	[0.10, 0.17]
Denmark	10.64	[0.08, 0.13]	Denmark	14.55	[0.12, 0.17]
Finland	12.28	[0.10, 0.15]	Finland	14.71	[0.12, 0.17]
Netherlands	13.59	[0.11, 0.16]	North Macedonia	18.29	[0.14, 0.23]
Spain	13.60	[0.12, 0.16]	Netherlands	18.48	[0.15, 0.22]
Switzerland	14.18	[0.11, 0.17]	Switzerland	18.78	[0.15, 0.23]
Slovenia	14.77	[0.13, 0.17]	Bosnia & Herzegovina	18.91	[0.14, 0.23]
Albania	15.21	[0.10, 0.20]	Slovenia	19.32	[0.17, 0.22]
Lithuania	15.61	[0.12, 0.19]	Albania	19.52	[0.13, 0.26]
Austria	15.76	[0.13, 0.18]	Austria	24.15	[0.21, 0.28]
Malta	15.84	[0.13, 0.19]	Norway	24.51	[0.22, 0.27]
Portugal	16.02	[0.13, 0.19]	Belgium	24.59	[0.23, 0.27]
Croatia	16.31	[0.13, 0.19]	Croatia	24.62	[0.21, 0.28]
Norway	17.00	[0.15, 0.19]	Malta	25.23	[0.22, 0.29]
Bosnia & Herzegovina	17.42	[0.14, 0.21]	Spain	21.66	[0.19, 0.24]
France	17.86	[0.15, 0.20]	Sweden	26.87	[0.23, 0.30]
Bulgaria	18.36	[0.15, 0.22]	Germany	26.80	[0.24, 0.29]
Luxembourg	19.00	[0.16, 0.22]	Bulgaria	27.10	[0.23, 0.31]
Belgium	19.01	[0.17, 0.21]	Lithuania	27.22	[0.23, 0.31]
North Macedonia	19.42	[0.15, 0.23]	Portugal	27.55	[0.24, 0.31]
Italy	19.70	[0.17, 0.22]	Montenegro	27.60	[0.23, 0.32]
Germany	20.41	[0.18, 0.22]	Luxembourg	28.08	[0.24, 0.32]
Poland	21.34	[0.18, 0.24]	Greece	28.66	[0.24, 0.33]
Sweden	21.61	[0.19, 0.25]	Italy	30.06	[0.27, 0.33]
Czechia	23.69	[0.20, 0.27]	Poland	31.06	[0.27, 0.35]
Montenegro	25.31	[0.21, 0.30]	France	31.24	[0.29, 0.34]
Serbia	25.42	[0.20, 0.31]	Serbia	30.69	[0.25, 0.36]
Cyprus	26.19	[0.22, 0.30]	Cyprus	32.70	[0.28, 0.37]
Greece	26.63	[0.23, 0.30]	Czechia	34.38	[0.31, 0.38]
Slovakia	34.00	[0.30, 0.38]	Slovakia	35.71	[0.32, 0.40]
United Kingdom	36.14	[0.33, 0.39]	United Kingdom	43.17	[0.40, 0.47]

Note: Analyses were performed adjusting for age and working hours. Countries are sorted by value of predicted probability. PP are displayed in %.

When grouping into regions and using the raw score, absolute frequencies of the raw score, similar absolute distribution pattern of all regions resulted (see Appendix Table 5 and Fig. 2). Overall, a unimodal, right-skewed distribution was observed. As WHO-5 scores increase, the proportion of individuals initially rises steadily, with the highest relative frequencies observed in the middle range (WHO-5: 14–20). Above this range, the proportions decline again at very high WHO-5 scores (WHO-5 ≥ 21).

We performed a robustness analysis including the Human Development Index on country level (results see in the Appendix in Table 8). The cross-level interaction between gender and the HDI was close to zero, indicating that the difference was robust across lower and higher HDI countries.

### Gender inequalities within different European regions and occupational groups

As shown in Table 3, the multilevel analysis, stratified first by region and then by occupational group, significant (*p* < .001) gender differences were observed across all subgroups, with odds ratios (ORs) varying by region and occupation.

The highest gender differences were found in Southern Europe, where women had 60% higher odds of high psychological distress relative to men, followed by Western (57% higher odds), then Northern (45% higher odds), then Eastern European countries where women had 39% higher odds of high psychological distress.

Regarding occupational groups, the largest gender difference appeared in the blue-collar low-skilled category, where women had 57% higher odds of psychological distress, followed by white-collar high-skilled (52% higher odds), then white-collar low-skilled (47% higher odds), then blue-collar high skilled (30% higher odds).

As detailed in Table 6 of the Appendix, analyses conducted without applying weights yielded slightly weaker effects but maintained a comparable overall pattern. Further, the analysis was robust to excluding covariates as seen in Table 7 in the Appendix.

**Table 3:** OR of high psychological distress among women in comparison to men by regions and occupation groups.

	OR	95% CI	*P*	PP Male	PP Female
Eastern Europe	1.39	[1.28, 1.50]	< 0.001	12.99	17.18
Northern Europe	1.45	[1.34, 1.56]	< 0.001	12.75	17.45
Southern Europe	1.60	[1.48, 1.73]	< 0.001	12.85	19.08
Western Europe	1.57	[1.45, 1.69]	< 0.001	11.64	17.10
White-collar high-skilled	1.52	[1.45, 1.60]	< 0.001	12.11	17.33
White-collar low-skilled	1.47	[1.35, 1.59]	< 0.001	15.27	20.89
Blue-collar high-skilled	1.30	[1.10, 1.53]	< 0.001	14.88	18.48
Blue-collar low-skilled	1.57	[1.38, 1.79]	< 0.001	13.68	19.92

Note: Multilevel logistic regression analyses were performed adjusting for age and working hours. PP are displayed in %.

The multilevel analysis including the interaction term region × occupational group (collar) demonstrated a significant interaction effect (*p* < .001). This indicates that the interaction of the variables adds information about the gender inequalities beyond the influence of the single variables alone.

**Fig. 1:**
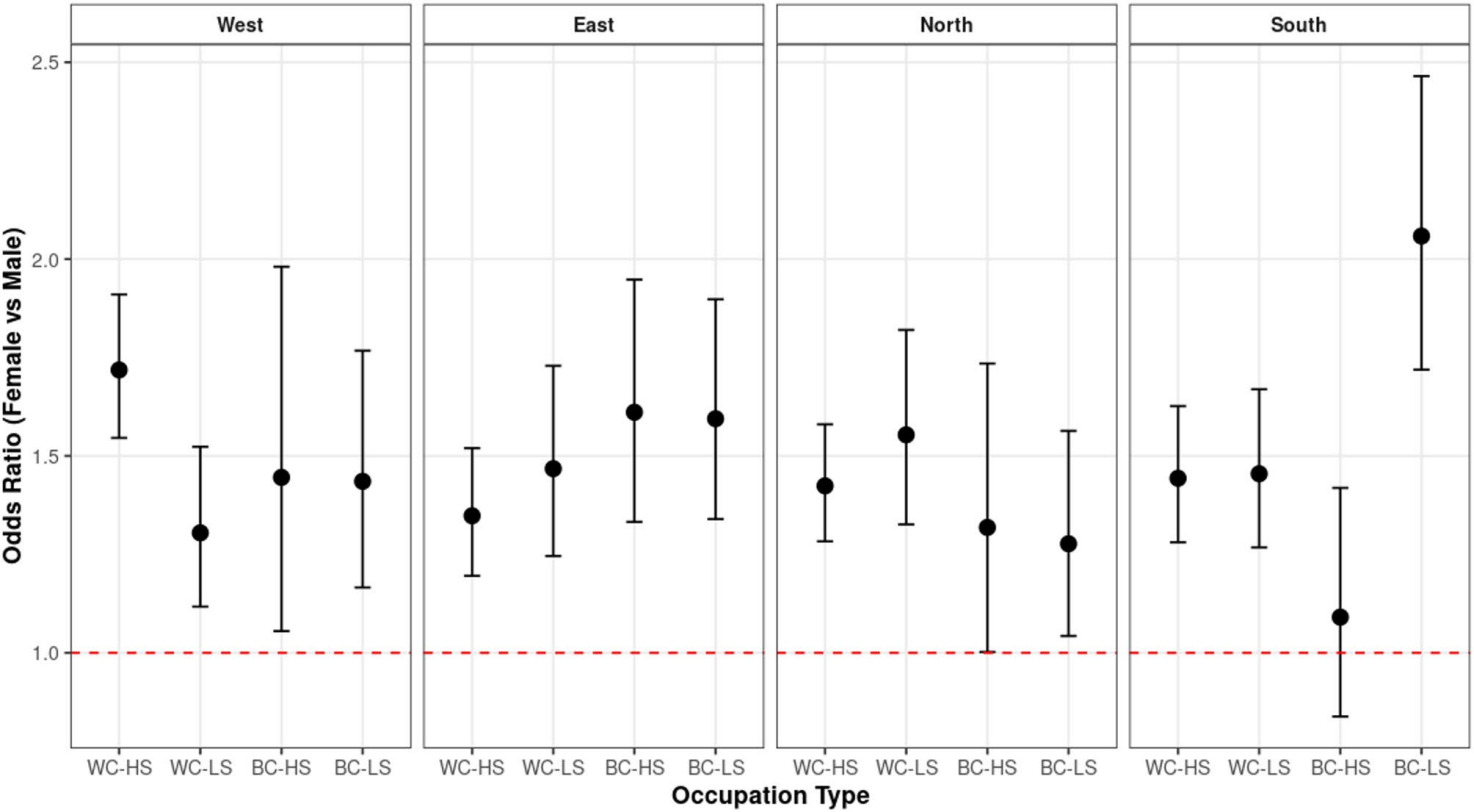
OR for high psychological distress among women in comparison to men. Note: Multilevel analysis with interaction region x collar. Analysis was performed adjusting for age and working hours. WC-HS = White-Collar High Skilled; WC-LS = White-Collar Low-Skilled; BC-HS = Blue-Collar High-Skilled; BC-LS = Blue-Collar Low-Skilled.

**Fig. 2:**
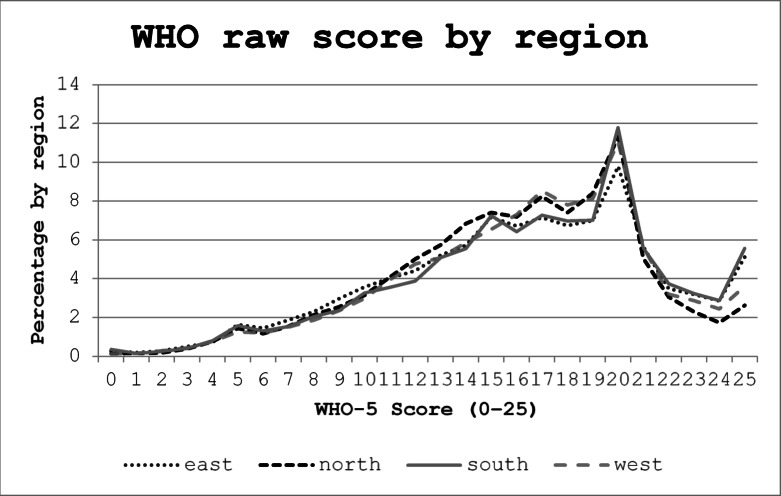
Line chart of frequencies of WHO5-raw-score by region over all genders.

**Fig. 3:**
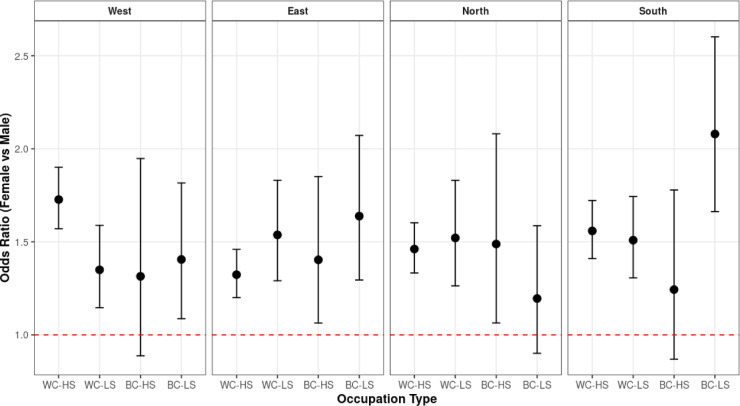
Multilevel analysis with interaction region x collar without weighting. Note: Depicted are odds ratios for high psychological distress. Analysis was performed adjusting for age and working hours. WC-HS = White-Collar High Skilled; WC-LS = White-Collar Low-Skilled; BC-HS = Blue-Collar High-Skilled; BC-LS = Blue-Collar Low-Skilled.

As illustrated in Fig. 1, the greatest gender inequalities were observed among white-collar high-skilled workers in Western Europe and blue-collar low-skilled workers in Southern Europe. Figure 3 in the Appendix shows that these central trends remain consistent when analyses are conducted without weighting the data.

## Discussion

Our research aimed to compare high psychological distress across multiple European regions and occupational groups. This study is among the first to simultaneously analyse several European regions using a consistent operationalization while incorporating occupational group distinctions.

### Key results

Regarding research question 1, substantial gender differences in high psychological distress with women experiencing greater burden than men were observed across all occupational groups and in most European countries, including Greece, Hungary, Slovenia, Bulgaria, Serbia, the Netherlands, the United Kingdom, Denmark, Belgium, Czechia, Germany, Cyprus, Sweden, Romania, Norway, Croatia, Ireland, Switzerland, Austria, Spain, Luxembourg, Poland, Italy, Lithuania, France, Malta, and Portugal.

Regarding research question 2, we observed variation in the magnitude of gender differences in high psychological distress across countries and the regions of Northern, Western, Southern, and Eastern Europe. Northern European countries exhibited the smallest gender differences, while Southern European countries showed the largest inequalities. However, since most confidence intervals overlapped, not all differences can be considered statistically or practically meaningful.

Although the values show considerable variability across countries, those with the highest and lowest levels of psychological distress are similar for both men and women with Kosovo, Romania and Denmark having particularly low values and United Kingdom, Slovakia and Serbia having particularly high values. Interestingly, no clear relationship emerges between the overall level of distress within a country and the magnitude of its gender differences, suggesting that these may be distinct phenomena or that unmeasured covariates could be influencing the observed patterns.

Regarding research question 3, gender differences in high psychological distress, stratified by occupational subgroup across Europe, were most pronounced in blue-collar low-skilled and white-collar high-skilled occupations. However, due to overlapping confidence intervals, these results should be interpreted with caution.

Regarding research question 4, the combination of blue-collar low-skilled occupations in Southern Europe and white-collar high-skilled occupations in Western Europe exhibited particularly large gender differences. These groups may be considered “vulnerable populations” with respect to pronounced gender inequalities in psychological distress and warrant further investigation in future research.

### Comparison with previous studies

The observed gender differences in high psychological distress align with prior research from various national and Europe-wide samples, consistently showing women to be more affected by distress or depression^4–7^. Van de Velde et al.^10^ reported modest region-specific patterns, with Eastern and Southern European countries exhibiting the largest gender gaps, whereas Schmitz and Brandt^12^ found somewhat differing regional trends, noting smaller gaps in Southern Europe. However, these regional distinctions are not sharply defined. Importantly, these prior studies primarily assessed diagnosed depression rather than high psychological distress, limiting direct comparability with our findings.

Our analyses indicated that Northern European countries tend to show relatively smaller gender inequalities, although overlapping confidence intervals suggest caution in interpreting country-specific differences. In their meta-analysis, Salk et al.^27^ distinguished between cross-national gender inequalities in clinical depression diagnoses and depressive symptoms without clinical diagnosis. Contrary to our results, they found gender differences predominantly in diagnosed clinical depression, but not consistently for symptoms alone. This discrepancy may stem from our use of a cut-off for high psychological distress, which was not applied in the meta-analysis. Additionally, Salk et al.^27^ observed somewhat higher gender-related odds ratios in high-income countries, though they cautioned these findings due to overlapping confidence intervals; these differences were more pronounced for clinical depression than for depressive symptoms. This aligns with our data, where some high-income countries show higher odds ratios, but not uniformly. The narrower range of odds ratios reported by Salk et al. compared to ours is consistent with our broader definition of high psychological distress as a screening tool for depression risk.

Regarding occupational differences, our results support findings from German data^4^, identifying white-collar high-skilled occupations as having notably large gender inequalities. While blue-collar low-skilled occupations were less emphasized in that study; Germany’s classification within Western Europe corresponds with the general trends observed in our analysis.

### Potential explanations for country-specific and region-specific gender inequalities

A potential explanatory factor is gender equality. Van de Velde et al.^10^ analysed a Europe-wide dataset from 2006/2007 and found that higher gender equality, measured by gender empowerment, was associated with smaller gender differences in depression, although this effect was specific to certain social subgroups. Similarly, Torsheim et al.^28^ reported that gender inequalities in general health complaints, including psychosomatic symptoms, were negatively correlated with the Gender Development Index in a European and North American adolescent sample from 1997/1998. However, these datasets are considerably older than ours, and cohort effects must be considered. Interestingly, Van de Velde et al.^10^ also observed that higher gender equality was linked to overall depression rates in a country, suggesting a relationship between gender equality and both depression prevalence and gender inequalities— a pattern we did not observe.

In the robustness analysis including the Human Development Index on country level, the cross-level interaction between gender and the HDI was close to zero. Therefore, the difference was robust across lower and higher HDI countries, and its components of life expectancy and average years of education seem to not sufficiently explain the gender inequalities in Europe.

By considering both societal context and individual circumstances, it may be possible to better understand why some countries exhibit wider gender disparities in psychological distress than others. In economically stronger countries, increased pressure to perform and challenges related to balancing work and family life, especially for women, may contribute to greater distress. Conversely, in other regions, more traditional gender roles or differing perceptions and reporting of mental health issues might influence results. Additionally, the availability of support systems, such as childcare institutions like kindergartens or all-day schools, child-friendly environments, and family assistance, varies widely between countries and can be influenced by cultural attitudes toward gender role division. For example, the availability, cost (or free provision), and extent of daycare for young children before school entry vary considerably by country and even within countries with federal systems, such as Germany. Therefore, in countries where the gender care gap exists at a bigger extent, gender inequalities in psychological distress might be further increased.

Moreover, countries differ in their occupational sector distributions, which involve varying workloads and resources. The gender composition within these sectors may affect gender differences in work-related stress. The impact of working hours, which we controlled for, might also differ depending on a country’s childcare system. The extent of role overload, particularly among part-time workers and women, may vary depending on childcare availability and societal norms.

### Partial explanations for occupation-specific gender inequalities

As a first explanation, the gender pay gap and the lack of feeling appropriately rewarded for one’s work might play a role. It has diminished over recent decades, but the reduction has been predominantly less pronounced in high-skilled and high-income jobs^29^. When feeling inadequately rewarded (e.g., through salary) for one’s work, psychological distress can increase, as described in the effort-reward imbalance model (ERI^30^), which is widely found to be valid^31^. As white-collar high-skilled occupations are most likely to be affected by the uneven gender pay gap trend, the ERI might partially explain why the white-collar high-skilled subgroup showed particularly high gender inequalities in high psychological distress. However, this line of argument does not match the big inequalities in the blue-collar low-skilled occupations.

As a second explanation, the exposure to different tasks and stresses may play a role. In a German employment survey reported by the Federal Institute for Occupational Health and Safety^32^ women were more likely than men to report multitasking, monotony, work interruptions, having to work quickly, being affected by emotionally stressful situations, as well as working at the limits of their capacity and in limited contracts. At the same time, women more frequently reported protective factors such as more freedom to plan and organize their work and more social support. It is possible that the reported stresses might be related to higher psychological distress in general or have a gender-specific effect. In a related German survey^14^, white-collar occupations were more associated with psychosocial and emotional stresses compared to blue-collar occupations. Therefore, it is possible that gender- and occupation-specific stresses interact with the countries’ labour laws and labour culture resulting in a complex mix of factors on distress on the genders in the occupations. Additionally, the proportion of people working in a different occupational group may vary by country and therefore influence the effect of each country. To control for that, latent class analysis to examine different country-group patterns with respect to high psychological distress among different occupational subgroups is needed in further research.

Further, gender imbalance can work as an additional stress factor. Although in the occupational subgroup of white-collar high-skilled the gender balance was nearly evenly distributed in the sample, the blue-collar low-skilled was male-dominated with a 1:5 ratio. Lacking female colleagues as social support and facing sexism and prejudices might increase psychological distress in these occupations for women additionally.

In our study, we used self-attributed gender as the independent variable. As described by de Breij et al.^33^, there remains a strong association between biological sex and stereotypical gender roles in the workplace. When analysing a large Dutch sample of older workers, participants registered as “female” scored higher on the gender index introduced by Smith and Koehoorn^34^. De Breij et al. found that female participants were more likely to work under stereotypically female conditions, including fewer working hours, higher levels of informal caregiving, more time spent on household chores, a higher proportion of women in their sector, lower educational attainment, and lower income indicating a systematic gender segregation at work. Additionally, the effect of gender index score on depressive symptoms was found to be even stronger than that of sex. This demonstrates that while sex can serve as a proxy for gender, including both biological sex and gender as a social construct in analyses can provide a more nuanced understanding of working conditions and structural inequalities, which is valuable for further research.

### Implications and relevance for the population

Psychological distress is a strong predictor of work incapacity and absenteeism and crossing the threshold into clinical depression significantly increases the risk of early exit from the workforce^1,2^. In a European wide comparison, it was found that women call in sick for work more often than men^35^. Especially in countries with weaker employees’ rights and social security systems, women’s additional load of financial loss due to absence or early work exit can aggravate gender inequalities in terms of financial security and independence. Research linking gender equality indices to disparities in psychological burden^28,36^ suggests that increased female representation in economic and political decision-making may be an effective strategy to incorporate perspectives that support female mental health and reduce gender-specific inequalities.

### Strengths and limitations

The major strength of this study lies in its cross-national design, which incorporates a nuanced perspective on occupational groups and includes a large number of countries using a consistent operationalization, thereby enabling valid comparisons of odds ratios. Converting the raw WHO-5 scores into a binary indicator of high psychological distress facilitates an application-oriented understanding of the clinically relevant implications of the data. However, this dichotomization entails a loss of some data granularity. Although the questionnaire was validated as a suitable screening tool for depression, every individual might experience levels of distress subjectively different in its level of restriction for their work, relationships or overall happiness. Therefore, crossing the threshold of “high distress” can impact individuals differently. Notably, when regression analyses were performed using the raw WHO-5 scores as the outcome variable, the main findings remained consistent.

The reliability and validity of self-reported symptoms can differ. Indeed, Topp et al.^16^ evaluated the validity of the WHO5 as a suitable screening tool in their literature review. The authors conclude few translation problems, that the questions do not seem to transgress cultural norms in the individual countries and assess high clinometric validity. Thus, self-assessing whether one feels rested upon waking might be easier than determining if one’s life is filled with things of interest, which demands more introspection and perceptual acuity (such as the ability to identify what interests one in general). This might also be influenced by cultural norms about what might be experienced and described as a psychological burden. Correspondingly, in a validation done by Sischka et al.^37^, the items were found to differ in difficulty and discrimination. Therefore, the authors conclude that the score method can lead to errors of inference. Although interviewers were trained and adequately compensated, they were not clinicians, which could impact validity due to variability in interviewing styles.

The chosen grouping of countries using the UN geoscheme might disregard regional differences that do not fit into its model. An alternative approach to grouping countries is to use the cultural dimensions described by Hofstede^38^. For example, Arrindell et al.^39^ found higher levels of depressiveness in nations with higher scores on Hofstede’s national masculinity index and lower levels of national wealth. Malik and Huo^40^ reported that higher levels of Hofstede’s individualism dimension and lower scores on the national masculinity index weakened the relationship between work stress and depressiveness. European regions could be grouped according to these indexes to examine whether not only overall distress, but also gender inequalities in distress, are associated with them.

A key limitation of this study is that data were collected in 2021, during the ongoing COVID-19 pandemic and associated lockdown measures. Consequently, our findings may reflect not only typical patterns but also the exceptional circumstances of that period. Variations in the severity of restrictions and the health situation across countries could have affected the absolute prevalence of high psychological distress, influencing cross-country and cross-regional comparisons. Conversely, pandemic-related restrictions may have somewhat homogenized working conditions, potentially attenuating gender inequalities. Future research using data from the post-pandemic period is needed to determine whether these observed effects persist under more typical conditions.

As the data relied on self-reported measures of experience of symptoms of distress, recall bias and social desirability bias as discussed by Bauhoff^41^ regarding self-reported health data might influence answering tendencies.

Participants identifying as “other” were excluded from the analysis due to their very small number. This highlights an ongoing gap in mental health research concerning individuals outside the gender binary – a group more frequently exposed to bullying^42^, which may affect workplace mental health and vary across countries and occupations.

Further, gender response bias might influence answering patterns. Male respondents might underscore on questions contradicting male gender norms and masculine ideals as not being active. Therefore, gender differences might even be underestimated.

Lastly, the dataset used in this study originates from a survey with a generally low response rate, which limits generalizability due to potential selection and non-response bias. Tolonen et al.^43^ found that about half of the non-participation in their Survey was attributed to “unsuitable timing and location.” In our context, individuals with occupations or caregiving responsibilities that allow greater flexibility are likely overrepresented. Moreover, because Eurofound and Ipsos contacted participants randomly and anonymously by telephone, it is unlikely that strategies recommended by Groves^44^, such as targeted recruitment, personalized communication, multiple contact attempts, pre-notification, or incentives, could be effectively implemented.

### Conclusion and opportunities for further research

Our findings show that the extent of gender differences in psychological distress varies across European regions and occupational groups. However, the underlying mechanisms behind these country-specific patterns remain largely unclear. One possible explanation is the role of working conditions and their gendered distribution, which have been shown to strongly influence mental health^42^. Future research could benefit from including work-related stressors to identify national differences in work culture and conditions that may contribute to observed gender disparities. Moreover, economic strength and gender equality at the national level should be further investigated for their potential interaction with gender inequalities in mental health. Despite the need for continued research, our findings offer robust evidence that gender inequalities in psychological distress remain a persistent public health issue in Europe, warranting targeted interventions adapted to regional and occupational contexts.

## Data Availability

The dataset employed in this study originates from the 2021 wave of the European Working Conditions Survey (EWCS), commissioned by Eurofound and conducted by Ipsos. Thus, the data cannot be distributed to the journal as the authors are not the owners of the data but only users. The dataset is freely available for academic purposes in a public repository on request. To apply for data access, contact the Head of Resources of Eurofound by mail (European Foundation for the Improvement of Living and Working Conditions / Eurofound​, ​Wyattville Road, Loughlinstown, Co. Dublin, D18 KP65, Ireland) or email (publicaccess@eurofound.europa.eu).

## Appendix 1

See Tables 4, 5, 6, 7 and 8; Figs. 2 and 3.

**Table 4:** Sensitivity analysis: predicted probabilities (PP) without covariates.

Country	PP	95% CI	Country	PP	95% confidence interval
Kosovo	0.0814	[0.06, 0.11]	Kosovo	0.1053	[0.06, 0.15]
Romania	0.104	[0.08, 0.13]	Romania	0.1386	[0.11, 0.17]
Denmark	0.1058	[0.08, 0.13]	Denmark	0.1421	[0.12, 0.17]
Finland	0.1235	[0.10, 0.15]	Finland	0.1443	[0.12, 0.17]
Netherlands	0.136	[0.11, 0.16]	Netherlands	0.1777	[0.15, 0.21]
Spain	0.1363	[0.12, 0.16]	Switzerland	0.181	[0.14, 0.22]
Switzerland	0.1436	[0.11, 0.17]	North Macedonia	0.1866	[0.14, 0.23]
Estonia	0.1466	[0.12, 0.18]	Estonia	0.1874	[0.16, 0.22]
Slovenia	0.1488	[0.13, 0.17]	Slovenia	0.1911	[0.17, 0.21]
Lithuania	0.1591	[0.12, 0.19]	Bosnia & Herzegovina	0.1933	[0.15, 0.24]
Albania	0.1611	[0.11, 0.21]	Albania	0.2039	[0.14, 0.27]
Austria	0.1614	[0.13, 0.19]	Spain	0.2118	[0.19, 0.24]
Portugal	0.1621	[0.14, 0.19]	Austria	0.2363	[0.20, 0.27]
Malta	0.1644	[0.14, 0.19]	Belgium	0.2412	[0.22, 0.26]
Norway	0.1697	[0.15, 0.19]	Norway	0.242	[0.21, 0.27]
Croatia	0.1698	[0.14, 0.20]	Croatia	0.25	[0.22, 0.28]
France	0.1807	[0.16, 0.20]	Malta	0.2593	[0.22, 0.30]
Bosnia & Herzegovina	0.1812	[0.14, 0.22]	Germany	0.2599	[0.24, 0.28]
Bulgaria	0.1876	[0.15, 0.22]	Latvia	0.2605	[0.23, 0.29]
Belgium	0.1923	[0.17, 0.21]	Sweden	0.2652	[0.23, 0.30]
Luxembourg	0.1962	[0.16, 0.23]	Lithuania	0.2668	[0.23, 0.30]
Italy	0.197	[0.17, 0.22]	Bulgaria	0.2681	[0.23, 0.31]
North Macedonia	0.2003	[0.16, 0.24]	Portugal	0.2731	[0.24, 0.31]
Germany	0.2058	[0.19, 0.23]	Montenegro	0.2825	[0.24, 0.33]
Sweden	0.2146	[0.18, 0.25]	Luxembourg	0.2845	[0.24, 0.33]
Poland	0.2202	[0.19, 0.25]	Greece	0.2867	[0.24, 0.33]
Czechia	0.2425	[0.21, 0.28]	Italy	0.2920	[0.26, 0.32]
Ireland	0.2521	[0.22, 0.28]	Serbia	0.3100	[0.26, 0.36]
Serbia	0.2653	[0.21, 0.32]	France	0.3103	[0.28, 0.34]
Montenegro	0.2655	[0.22, 0.31]	Poland	0.3138	[0.28, 0.35]
Latvia	0.2689	[0.23, 0.31]	Cyprus	0.3347	[0.29, 0.38]
Cyprus	0.2697	[0.23, 0.31]	Czechia	0.3395	[0.30, 0.37]
Greece	0.2761	[0.24, 0.32]	Hungary	0.3406	[0.30, 0.38]
Hungary	0.2981	[0.26, 0.34]	Ireland	0.3414	[0.30, 0.38]
Slovakia	0.3492	[0.31, 0.39]	Slovakia	0.3571	[0.32, 0.40]
United Kingdom	0.3668	[0.33, 0.40]	United Kingdom	0.4274	[0.39, 0.46]

**Table 5:** Frequencies of WHO5-raw-score by region in percent over all genders.

WHO5score	Eastern Europe	Northern Europe	Southern Europe	Western Europe
0	0.28	0.14	0.35	0.13
1	0.19	0.16	0.15	0.16
2	0.29	0.18	0.25	0.29
3	0.50	0.39	0.40	0.44
4	0.72	0.76	0.80	0.76
5	1.64	1.44	1,57	1.26
6	1.46	1.17	1.30	1.20
7	1.87	1.54	1.54	1.51
8	2.30	2.14	2.05	1.87
9	2.97	2.55	2.37	2.35
10	3.57	3.15	3.26	2.98
11	4.02	4.08	3.55	3.95
12	4.41	5.01	3.88	4.76
13	5.21	5.74	5.09	5.08
14	5.72	6.84	5.55	5.86
15	7.18	7.40	7.24	6.54
16	6.71	7.19	6.42	7.30
17	7.15	8.24	7.27	8.53
18	6.74	7.41	6.97	7.81
19	7.00	8.39	7.01	8.13
20	9.78	11.32	11.78	11.04
21	5.65	5.04	5.58	5.64
22	3.49	3.06	3.74	3.20
23	3.19	2.29	3.24	2.87
24	2.85	1.74	2.87	2.45
25	5.12	2.63	5.56	3.62

**Table 6:** Multilevel logistic regression without weights.

Subgroup	OR	*P*	95% CI
Eastern Europe	1.39	< 0.001	[1.28, 1.50]
Northern Europe	1.45	< 0.001	[1.34, 1.56]
Southern Europe	1.60	< 0.001	[1.48, 1.73]
Western Europe	1.57	< 0.001	[1.45, 1.69]
White-collar high-skilled	1.52	<0.001	[1.45, 1.60]
White-collar low-skilled	1.47	< 0.001	[1.35, 1.59]
Blue-collar high-skilled	1.30	< 0.001	[1.10, 1.53]
Blue-collar low-skilled	1.57	< 0.001	[1.38, 1.79]

Note: Depicted are the odds ratios for high psychological distress. Analyses were performed adjusting for age and working hours.

**Table 7:** Sensitivity analysis with OR of high psychological distress among women compared to men without covariates (only gender).

Subgroup	OR	95% CI	*P*	PP Men	PP Women
Eastern Europe	1.32	[1.23, 1.43]	< 0.001	14.13	17.90
Northern Europe	1.39	[1.29, 1.50]	< 0.001	13.93	18.39
Southern Europe	1.51	[1.40, 1.62]	< 0.001	14.32	20.15
Western Europe	1.52	[1.42, 1.64]	< 0.001	12.09	17.34
White-collar high-skill	1.46	[1.39, 1.54]	<0.001	12.78	17.66
White-collar low-skill	1.42	[1.30, 1.54]	< 0.001	14.38	19.22
Blue-collar high-skill	1.26	[1.07, 1.49]	0.007	15.55	18.80
Blue-collar low-skill	1.30	[1.30, 1.67]	< 0.001	14.35	19.79

Note: PP are displayed in %.

**Table 8:** Sensitivity analysis including Human Development Index (HDI).

Predictor	Coefficient	*P*	OR	95% CI
Female (vs. male)	0.39	<0.001	1.48	1.40; 1.56
HDI	-0.07	0.22	0.93	0.84; 1.04
Gender × HDI	-0.003	0.90	0.996	0.95; 1.05
Age in years	-0.01	<0.001	0.99	0.99; 0.99
Usual weekly hours	0.01	<0.001	1.01	1.01; 1.01

Note: Calculation via Mixed-effects logistic regression analysis. DV= high psychological distress (yes/no), IV= gender, adjusting for age and usual weekly working hours. Random intercepts and random slopes for gender were specified at the country level to allow both baseline distress levels and the gender gap to vary across countries. Country-level HDI- values were z-standardized and entered as a level 2 predictor, along with a cross-level interaction (gender × HDI) to test whether the gender gap differed by national HDI-value.

## References

[1] Duijts, S. F. A., Kant, I., Swaen, G. M. H., Van Den Brandt, P. A. & Zeegers, M. P. A. A meta-analysis of observational studies identifies predictors of sickness absence. *J. Clin. Epidemiol.* **60**, 1105–1115 (2007). 17938051 10.1016/j.jclinepi.2007.04.008

[2] Borritz, M., Rugulies, R., Christensen, K. B., Villadsen, E. & Kristensen, T. S. Burnout as a predictor of self-reported sickness absence among human service workers: prospective findings from three year follow up of the PUMA study. *Occup. Environ. Med.* **63**, 98–106 (2006). 16421387 10.1136/oem.2004.019364 PMC2078067

[3] Viertiö, S. et al. Factors contributing to psychological distress in the working population, with a special reference to gender difference. *BMC Public. Health*. **21**, 611 (2021). 33781240 10.1186/s12889-021-10560-y PMC8006634

[4] Grasshoff, J., Safieddine, B., Sperlich, S. & Beller, J. Gender inequalities of psychosomatic complaints at work vary by occupational groups of white- and blue-collar and level of skill: A cross sectional study. *PLoS ONE*. **19**, e0303811 (2024). 38990805 10.1371/journal.pone.0303811 PMC11239076

[5] Hünefeld, L. & Dötsch, M. Berufliche Geschlechtersegregation. Die Arbeits- und Gesundheitssituation von Frauen und Männern in geschlechtersegregierten und -integrierten Berufen. (2023). 10.21934/BAUA:FOKUS20230324

[6] Campos-Serna, J., Ronda-Pérez, E., Artazcoz, L., Moen, B. E. & Benavides, F. G. Gender inequalities in occupational health related to the unequal distribution of working and employment conditions: a systematic review. *Int. J. Equity Health*. **12**, 57 (2013). 23915121 10.1186/1475-9276-12-57 PMC3765149

[7] Campaña, J. C., Gimenez-Nadal, J., Velilla, J. & & Measuring Gender Gaps in Time Allocation in Europe. *Soc. Indic. Res.* **165**10.1007/s11205-022-03026-0 (2022).

[8] Gencer, H., Brunnett, R., Staiger, T., Tezcan-Güntekin, H. & Pöge, K. Caring is not always sharing: A scoping review exploring how COVID-19 containment measures have impacted unpaid care work and mental health among women and men in Europe. *PLoS ONE*. **19**, e0308381 (2024). 39213370 10.1371/journal.pone.0308381 PMC11364293

[9] Kjellsson, S. Do working conditions contribute differently to gender gaps in self-rated health within different occupational classes? Evidence from the Swedish Level of Living Survey. *PLoS One*. **16** (6), e0253119. 10.1371/journal.pone.0253119 (2021). 34129618 10.1371/journal.pone.0253119 PMC8205134

[10] Van De Velde, S., Huijts, T., Bracke, P. & Bambra, C. Macro-level gender equality and depression in men and women in Europe. *Sociol. Health Illn.* **35**, 682–698 (2013). 23145770 10.1111/j.1467-9566.2012.01521.x

[11] Herlitz, A., Hönig, I., Hedebrant, K. & Asperholm, M. A Systematic Review and New Analyses of the Gender-Equality Paradox. *Perspect. Psychol. Sci.* **20**, 503–539 (2025). 38170215 10.1177/17456916231202685 PMC12065958

[12] Schmitz, A. & Brandt, M. Gendered patterns of depression and its determinants in older Europeans. *Arch. Gerontol. Geriatr.* **82**, 207–216 (2019). 30831527 10.1016/j.archger.2019.02.015

[13] Lahti, J., Knop, J., Lallukka, T., Harkko, J. & Kouvonen, A. Occupational Class Differences in Emotional Exhaustion Among Municipal Employees – The Role of Employment Sector and Psychosocial Working Conditions. *Psychol. Rep.* **126**, 3104–3122 (2023). 35642717 10.1177/00332941221106393 PMC10652648

[14] Hämmig, O. & Bauer, G. F. The social gradient in work and health: a cross-sectional study exploring the relationship between working conditions and health inequalities. *BMC Public. Health*. **13**, 1170 (2013). 24330543 10.1186/1471-2458-13-1170 PMC4028882

[15] Eurofound, I. N. V. & Belgium *Working Conditions and Sustainable Work. European Working Conditions Telephone Survey 2021: Technical Report.* https://www.eurofound.europa.eu/system/files/2022-12/wpef22050.pdf (2021).

[16] Topp, C. W., Østergaard, S. D., Søndergaard, S. & Bech, P. The WHO-5 Well-Being Index: A Systematic Review of the Literature. *Psychother. Psychosom.* **84**, 167–176 (2015). 25831962 10.1159/000376585

[17] Nylén-Eriksen, M. et al. Validating the Five-Item World Health Organization Well-Being Index. *IJERPH* **19**, 11489 (2022). 36141760 10.3390/ijerph191811489 PMC9517039

[18] Massing, N., Wasmer, M., Wolf, C. & Zuell, C. How Standardized is Occupational Coding? A Comparison of Results from Different Coding Agencies in Germany. *J. Official Stat.* **35**, 167–187 (2019).

[19] European Foundation for the Improvement of Living and Working Conditions. & European Commission. Joint Research Centre. European Jobs Monitor 2021: Gender Gaps and the Employment Structure. (Publications Office, LU, (2021).

[20] Beller, J., Graßhoff, J. & Safieddine, B. Physical working conditions over time: a repeated cross-sectional study in German employees. *J. Occup. Med. Toxicol.* **19**, 24 (2024). 38858744 10.1186/s12995-024-00423-8 PMC11165766

[21] Beller, J. et al. Decline of depressive symptoms in Europe: Differential trends across the lifespan. Social Psychiatry and Psychiatric Epidemiology. (2021). 10.1007/s00127-020-01979-6 10.1007/s00127-020-01979-6 PMC8225536 33180149

[22] Oshio, T. Evolution of psychological distress with age and its determinants in later life: evidence from 17-wave social survey data in Japan. *BMC Public. Health*. **24**10.1186/s12889-024-19912-w (2024). 10.1186/s12889-024-19912-w PMC11367901 39223518

[23] Ganster, D. C., Rosen, C. C. & Fisher, G. G. Long Working Hours and Well-being: What We Know, What We Do Not Know, and What We Need to Know. *J. Bus. Psychol.* **33**, 25–39 (2018).

[24] Yoon, Y., Ryu, J., Kim, H., Kang, C. W. & Jung-Choi, K. Working hours and depressive symptoms: the role of job stress factors. *Ann. Occup. Environ. Med.* **30**, 46 (2018). 30009036 10.1186/s40557-018-0257-5 PMC6043993

[25] Artazcoz, L. et al. Long working hours and health in Europe: Gender and welfare state differences in a context of economic crisis. *Health Place*. **40**, 161–168 (2016). 27341274 10.1016/j.healthplace.2016.06.004

[26] United Nations. Standard country or area codes for statistical use (M49). https://unstats.un.org/unsd/methodology/m49/ (n.d.).

[27] Salk, R. H., Hyde, J. S. & Abramson, L. Y. Gender differences in depression in representative national samples: Meta-analyses of diagnoses and symptoms. *Psychol. Bull.* **143**, 783–822 (2017). 28447828 10.1037/bul0000102 PMC5532074

[28] Torsheim, T. et al. Cross-national variation of gender differences in adolescent subjective health in Europe and North America. *Soc. Sci. Med.* **62**, 815–827 (2006). 16098649 10.1016/j.socscimed.2005.06.047

[29] Blau, F. D. & Kahn, L. M. The Gender Wage Gap: Extent, Trends, and Explanations. *J. Econ. Lit.* **55**, 789–865 (2017).

[30] Siegrist, J. Adverse health effects of high-effort/low-reward conditions. *J. Occup. Health Psychol.* **1**, 27–41 (1996). 9547031 10.1037//1076-8998.1.1.27

[31] Rugulies, R., Aust, B. & Madsen, I. E. Effort–reward imbalance at work and risk of depressive disorders. A systematic review and meta-analysis of prospective cohort studies. *Scand. J. Work Environ. Health*. **43**, 294–306 (2017). 28306759 10.5271/sjweh.3632

[32] Bundesanstalt für Arbeitsschutz und Arbeitsmedizin (BAuA). Stressreport Deutschland 2019: Psychische Anforderungen, Ressourcen Und Befinden. https://www.baua.de/DE/Angebote/Publikationen/Berichte/Stressreport-2019.html?pk_campaign=DOI (2020). 10.21934/BAUA:BERICHT20191007

[33] De Breij, S., Huisman, M., Boot, C. R. L. & Deeg, D. J. H. Sex and gender differences in depressive symptoms in older workers: the role of working conditions. *BMC Public. Health*. **22**, 1023 (2022). 35597949 10.1186/s12889-022-13416-1 PMC9123290

[34] Smith, P. M. & Koehoorn, M. Measuring gender when you don’t have a gender measure: constructing a gender index using survey data. *Int. J. Equity Health*. **15**, 82 (2016). 27233478 10.1186/s12939-016-0370-4 PMC4884354

[35] Eurofound. Working conditions and workers’ health. Publications Office of the European Union, Luxembourg. 10.2806/909840 (2019).

[36] Van de Velde, S., Huijts, T., Bracke, P. & Bambra, C. Macro-level gender equality and depression in men and women in Europe. *Sociol. Health Illn.* **35** (5), 682–698. 10.1111/j.1467-9566.2012.01521.x (2013). 23145770 10.1111/j.1467-9566.2012.01521.x

[37] Sischka, P. E., Costa, A. P., Steffgen, G. & Schmidt, A. F. The WHO-5 well-being index – validation based on item response theory and the analysis of measurement invariance across 35 countries. *J. Affect. Disorders Rep.* **1**, 100020 (2020).

[38] Hofstede, G. Culture’s Consequences: Comparing Values, Behaviors, Institutions and Organizations Across Nations. (2001). 10.1016/S0005-7967(02)00184-5

[39] Arrindell, W. A., Steptoe, A. & Wardle, J. Higher levels of state depression in masculine than in feminine nations. *Behav. Res. Ther.* **41**(7), 809 – 817.10.1016/s0005-7967(02)00185-7 (2003). 10.1016/s0005-7967(02)00185-7 12781247

[40] Malik, T. H. & Huo, C. National Cultural Moderates the Link Between Work Stress and Depression: An Analysis of Clinical Trial Projects Across Countries. *Cross-Cultural Res.* **57** (1), 23–55. 10.1177/10693971221131427 (2022). (Original work published 2023).

[41] Bauhoff, S. Systematic self-report bias in health data: impact on estimating cross-sectional and treatment effects. *Health Serv. Outcomes Res. Method*. **11**, 44–53 (2011).

[42] Artazcoz, L. et al. Socioeconomic characteristics, health and wellbeing of nonbinary adolescents in a Southern European City. *BMC Public. Health*. **25**, 1932 (2025). 40420040 10.1186/s12889-025-23080-w PMC12105259

[43] Tolonen, H., Lundqvist, A., Jääskeläinen, T., Koskinen, S. & Koponen, P. Reasons for non-participation and ways to enhance participation in health examination surveys—the Health 2011 Survey. *Eur. J. Pub. Health*. **27**, 909–911 (2017). 28957480 10.1093/eurpub/ckx098

[44] Groves, R. M. & Peytcheva, E. The Impact of Nonresponse Rates on Nonresponse Bias: A Meta-Analysis. *Pub. Opin. Q.* **72**, 167–189 (2008).

